# Obesity-focused prehabilitation strategies in ventral hernia: Cohort study

**DOI:** 10.1007/s10029-025-03392-x

**Published:** 2025-06-11

**Authors:** Víctor Rodrigues-Gonçalves, Mireia Verdaguer-Tremolosa, Pilar Martínez-López, Clara Nieto, Sana Khan, Manuel López-Cano

**Affiliations:** General Surgery Department, Abdominal Wall Surgery Unit, Hospital Universitari Vall d´Hebron, Universitat Autònoma de Barcelona, Paseo Vall d`Hebron 119-129, 08035 Barcelona, Spain

**Keywords:** Ventral hernia repair, Preoperative weight loss, Bariatric surgery, Surgery outcomes, Obesity

## Abstract

**Purpose:**

Obesity increases the risk of complications and technical difficulty in ventral hernia repair. Preoperative weight loss is recommended to mitigate these risks, but the implementation of different strategies in routine practice remains poorly described. This study aimed to characterize the use of dietary counseling, pharmacotherapy, and bariatric surgery within a structured optimization pathway and provide preliminary insights into surgical outcomes in obese versus non-obese patients.

**Methods:**

In this retrospective, single-center study, obese patients with ventral hernia were managed with dietary counseling, pharmacotherapy, or bariatric surgery between April 2018 and April 2023. We evaluated implementation, weight loss achieved, eligibility for elective repair, and adherence. Surgical outcomes were descriptively analyzed in obese patients with and without preoperative weight loss and compared to non-obese patients.

**Results:**

Of 175 obese patients, 148 (84.6%) received dietary counseling, 15 (8.6%) pharmacotherapy, and 12 (6.8%) bariatric surgery. Median weight loss was highest after bariatric surgery (20.7%), followed by dietary counseling (4.6%) and pharmacotherapy (4.4%). Surgical eligibility rates were 83%, 44%, and 13%, respectively. Among 165 patients who underwent hernia repair, postoperative complications were more frequent in obese patients, regardless of preoperative weight loss, than in non-obese patients. Recurrence was numerically higher in patients without preoperative weight loss, though not statistically significant.

**Conclusion:**

A structured optimization pathway facilitated the use of diverse weight loss strategies before hernia repair in obese patients. Bariatric surgery achieved the greatest weight loss and eligibility. However, complications remained common, underscoring the need for individualized, multidisciplinary prehabilitation strategies.

## Introduction

Ventral hernia is a common pathology that represents a significant challenge in surgical practice because of the high rate of recurrence and associated postoperative complications [1, 2]. In particular, obesity has been identified as an important risk factor that increases the likelihood of complications such as infections, wound dehiscence, and the need for reintervention [3, 4]. These complications not only affect patient recovery but also increase the costs associated with medical care and prolong hospitalization time [5–7].

Preoperative weight loss has been proposed as a strategy to mitigate the risks associated with obesity in patients undergoing elective ventral hernia surgery [8]. Several studies have suggested that a reduction in body mass index (BMI) before surgery may decrease the incidence of complications and improve surgical outcomes [9–11]. However, considerable variability in the strategies employed to achieve weight loss is noted, ranging from dietary modifications to more invasive procedures such as bariatric surgery [12]. Recently, weight loss pharmacotherapy has gained popularity as a treatment option for obesity [13]. Although the results of this strategy in the preoperative optimization of patients with ventral hernia seem promising [14], evidence regarding its effectiveness in this context remains limited. The comparative efficacy of these strategies in the context of ventral hernia surgery has not been fully elucidated, highlighting the need for further studies evaluating their impact.

This study aims to describe the implementation and application of three preoperative weight loss strategies (diet and exercise, pharmacotherapy, and bariatric surgery) in patients with obesity undergoing ventral hernia repair within a specialized abdominal wall unit. It also provides a preliminary assessment of surgical outcomes in these patients, compared with a cohort of non-obese patients operated on during the same period.

## Methods

### Study design

This was a retrospective cohort study conducted at the Abdominal Wall Surgery Unit of Vall d'Hebron University Hospital, accredited by the Spanish Association of Surgeons [15]. The study adhered to the principles outlined in the Declaration of Helsinki and followed the Strengthening the Reporting of Observational Studies in Epidemiology (STROBE) [16] and Reporting of Studies Conducted Using Observational Routinely Collected Health Data (RECORD) [17] guidelines. Data from patients who visited for ventral hernia with obesity between April 2018 and April 2023 were retrospectively gathered from a prospectively maintained database. This retrospective study was based on anonymized data collected during routine clinical practice and, in accordance with local regulations, did not require approval by a research ethics committee.

### Patients

The inclusion criteria were as follows: 1) aged ≥ 18 years and 2) patients who visited the specialized abdominal wall surgery unit for ventral hernia with a BMI ≥ 30 kg/m^2^. The exclusion criteria were as follows: 1) patients who visited other nonspecialized abdominal wall surgery units of the hospital for ventral hernia; 2) patients with a BMI ≥ 30 kg/m^2^ and had other hernias, such as inguinal femoral hernias; 3) patients with severe comorbidities, such as metastatic cancer or advanced cirrhosis, which are expected to limit survival to less than two years; and 4) pregnant patients.

In our unit, preoperative risk factors are routinely evaluated during the initial consultation, and a special emphasis is placed on obesity as a modifiable factor to improve surgical outcomes. Patients without significant risk factors, such as obesity, were considered suitable for elective surgery. Patients with a BMI ≥ 30 kg/m^2^ were enrolled in a weight loss program to optimize their condition prior to surgery. Surgery was postponed until adequate weight loss was achieved.

The management of patients with ventral hernia and obesity followed a structured institutional optimization pathway based on BMI thresholds and clinical criteria, in accordance with current evidence-based guidelines [12, 18]. This protocol standardized the use of dietary counseling, pharmacotherapy, or bariatric surgery as weight-loss interventions, and included regular follow-up visits every three months to monitor weight, BMI, symptoms, and adherence. For patients with a BMI between 30 and 35 kg/m^2^, dietary counseling was provided, with an emphasis on changing eating habits and engaging in regular physical activity. Additionally, the risks of obesity, both in general terms and specifically in relation to ventral hernia surgery, were thoroughly explained, and weight loss goals were established. These patients were also advised to visit their primary care physician for the optimization and management of any comorbid conditions, such as hypertension or diabetes. The objective was to achieve a BMI as close as possible to 30 kg/m^2^, with sustained weight loss before proceeding with elective hernia repair [18]. However, eligibility was assessed on an individualized basis, considering patient characteristics, hernia complexity, and symptom severity. Patients with a BMI ≥ 35 kg/m^2^ were referred to a comprehensive obesity treatment unit within the hospital. There, a multidisciplinary team composed of endocrinologists, nutritionists, psychologists, and metabolic surgeons assessed the patients to determine the most appropriate intervention, which could include dietary modifications, pharmacotherapy, or bariatric surgery, depending on the patient’s age, comorbidities, and personal preferences. For patients with BMI ≥ 40 kg/m^2^ or BMI ≥ 35 kg/m^2^ with significant obesity-related comorbidities, bariatric surgery was generally recommended, provided they met the necessary clinical and psychological criteria. Contraindications included severe cardiovascular disease, uncontrolled psychiatric disorders, active substance abuse, and the inability to adhere to postoperative dietary and lifestyle modifications. Patients deemed unsuitable for surgery were managed with dietary counseling and/or pharmacotherapy as alternative weight-loss strategies. These patients were required to achieve substantial and sustained weight loss before elective hernia repair was considered. For all patients, surgery was generally postponed until meaningful weight reduction was achieved, but exceptions were made for those with recurrent bowel obstruction, where delaying repair posed a higher risk. All patients were followed every three months, during which weight, BMI, and hernia symptoms were monitored. Emergency visits due to incarceration were also recorded, and the importance of adhering to the weight loss goals was reinforced.

For descriptive analysis, patients were grouped according to the recommended intervention within the structured pathway: (1) dietary and behavioral counseling, (2) pharmacological treatment for obesity, or (3) bariatric surgery. Each patient was assigned to a single group based on the initial weight-loss strategy recommended by the clinical team, following institutional protocols and considering patient preferences. Grouping was mutually exclusive for analysis purposes, and patients were not reassigned between strategies during follow-up. As this process involved shared decision-making, baseline characteristics such as age, BMI, and comorbidity burden differed across groups. In each group, the percentage of weight loss and percentage of BMI reduction, adherence to the weight loss protocol, incidence of urgent ventral hernia surgery, and rate of elective hernia surgery were documented. Surgical outcomes were also reported for patients who underwent hernia repair, including a descriptive comparison with non-obese patients treated during the same period.

### Study variables

Patient demographic and clinical variables were collected at the initial presentation. These included age, sex, and BMI at both the initial consultation and the final follow-up. The American Society of Anesthesiologists (ASA) classification was recorded, along with the presence of comorbidities. The comorbidities documented included cardiovascular disease, chronic obstructive pulmonary disease (COPD), diabetes, anticoagulant therapy, nephropathy, and active smoking. For patients who underwent ventral hernia surgery, additional data were gathered, including the patient’s age and BMI at surgery.

Hernia-related data included symptoms, defect size, complexity, and recurrence status. Hernia complexity was defined according to established criteria, including defect > 10 cm, loss of domain ≥ 20%, recurrent hernia, multiple defects, history of surgical site infections, need for component separation, wound class III–IV, skin grafts, and prior intraperitoneal mesh removal [19]. The type of hernia was classified into specific subtypes: umbilical, midline, trocar site, parastomal, combined, lateral, and suprapubic hernias. The recurrence of the hernia was also noted.

The operative records of patients who underwent ventral hernia repair were reviewed. Patients were classified into one of two groups on the basis of their adherence to preoperative weight loss recommendations: 1) patients who met the preoperative weight loss target and 2) patients who did not meet the preoperative weight loss goal but underwent surgery due to symptom severity. These two groups were compared to a control cohort of nonobese patients who underwent ventral hernia repair in our unit during the same period.

Patients were treated with an open, laparoscopic, or robotic approach. The choice of approach was at the discretion of the surgeon on the basis of his or her preference and patient characteristics. In general, techniques involving preperitoneal/retromuscular mesh placement are the preferred approach in our unit, in accordance with clinical guidelines for the treatment of ventral hernias [18, 20]. The decision regarding mesh placement (retromuscular, onlay, inlay or intraperitoneal) was based on patient-specific factors, including hernia size, location, previous surgical history, and surgeon preference, following clinical guidelines.

Additional operative variables included operative time, defined as the time from incision to the placement of the dressing, and whether component separation techniques were employed for hernia repair. Component separation was performed in cases where primary fascial closure was not feasible without excessive tension, particularly in patients with large defects, loss of domain, or recurrent hernias [20].

Postoperative variables included complications (within 90 days postoperatively), length of hospital stay (in days), and hernia recurrence. Postoperative complications included hematoma, seroma, wound infection, and surgical site ocurrences (SSO) [21]. The complications were classified according to the Clavien-Dindo grading system [22].

Hernia recurrence was assessed using a combination of clinical follow-up and patient-reported outcomes. Medical records were reviewed for any intervention due to hernia recurrence, either through surgical reintervention or diagnosis during physical examinations. Additionally, the Ventral Hernia Recurrence Inventory (VHRI) [23], a validated questionnaire specifically designed for ventral hernia patients [24], was used during telephone follow-ups to screen for recurrence. Any patient with a positive response on the VHRI was advised to schedule an in-person visit for physical examination. For patients who were lost to telephone follow-up, their last recorded in-person postoperative visit was used as the final follow-up date.

### Statistical analysis

Quantitative variables are reported as the means and standard deviations and were analyzed using Student's t test or the Mann‒Whitney U test when necessary. Qualitative variables are expressed as counts and percentages and were compared using the chi-square test or Fisher's exact test when necessary. A *P* value < 0.05 indicated statistical significance. SPSS (IBMS SPSS Statistics 23) was used for statistical analysis.

## Results

### Patient demographics and clinical characteristics of obese patients with ventral hernia

A total of 175 obese patients with ventral hernias were included in the study, of whom 148 (84.6%) underwent dietary counseling, 15 (8.6%) received pharmacotherapy, and 12 (6.8%) underwent bariatric surgery. The groups differed significantly in age distribution (*P* < 0.001): bariatric surgery patients were the youngest (mean 46 years, SD: 7.9), followed by the pharmacotherapy group (mean 57.5 years, SD: 10.7), while dietary counseling patients were the oldest (mean 61 years, SD: 12.6). No significant differences were observed in sex distribution (*P* = 0.592), comorbidities (*P* = 0.876), or hernia complexity (*P* = 0.364) (Table 1).

**Table 1:** Effects of weight loss strategies in patients with ventral hernia and obesity

Variable	Total (*n* = 175)	Dietary and behavioral counseling (*n* = 148)	Bariatric surgery (*n* = 12)	Pharmacotherapy (*n* = 15)	*P*
Age (yrs) [mean (SD)]	59.6 (12.8)	61 (12.6)	46 (7.9)	57.5 (10.7)	< 0.001
Gender, *n* (%)					0.592
Male	83 (47)	72 (49)	4 (33)	7 (47)	
Female	92 (53)	76 (51)	8 (67)	8 (53)	
ASA score					0.650
I/II [*n*, (%)]	111 (63)	92 (62)	9 (75)	10 (67)	
III [*n*, (%)]	64 (37)	56 (38)	3 (25)	5 (33)	
Previous abdominal surgery, *n* (%)	129 (74)	106 (72)	11 (92)	12 (80)	0.268
Comorbidity, *n* (%)	141 (81)	120 (81)	9 (75)	12 (80)	0.876
Cardiovascular disease, *n* (%)	108 (62)	93 (63)	6 (50)	9 (60)	0.672
Diabetes, *n* (%)	41 (23)	33 (22)	2 (17)	6 (40)	0.258
Comorbidity more than one, *n* (%)	85 (49)	73 (49)	4 (33)	8 (53)	0.526
Symptomatic hernia, n (%)	128 (73)	107 (72)	11 (92)	10 (67)	0.291
Recurrent hernia, *n* (%)	30 (17)	26 (18)	2 (17)	2 (13)	0.917
Complex hernia, *n* (%)	87 (50)	75 (51)	7 (58)	5 (33)	0.364
Initial weight (kg) [mean, (SD)]	96.8 (16.1)	95 (14.7)	116.9 (22.7)	99.1 (12)	< 0.001
Weight at last follow-up (kg) [mean, (SD)]	92.1 (16.7)	91 (14.7)	94.5 (32.5)	97.5 (16.8)	0.344
Percentage of weight loss [%, (SD)]	5.7 (7.9)	4.6 (6)	20.7 (15.5)	4.4 (3.5)	< 0.001
Initial BMI (kg/m^2^) [mean, (SD)]	36.2 (4.7)	35.5 (4.4)	42.8 (4.7)	37.9 (4.2)	< 0.001
BMI at last follow-up (kg/m^2^) [mean, (SD)]	34 (5.7)	33.9 (5.3)	33.9 (8.8)	36.9 (5.7)	0.143
Percentage of BMI loss [%, (SD)]	6.2 (8.3)	5 (6.4)	21.7 (15.5)	5.1 (4.9)	< 0.001
Ventral hernia surgery [%, (SD)]					0.001
No surgery	98 (56)	83 (56)	2 (17)	13 (87)	
Surgery	77 (44)	65 (44)	10 (83)	2 (13)	
Abandonment of weight loss program [%, (SD)]	33 (19)	31 (21)	0 (0)	2 (13)	0.173

*ASA* American Society of Anesthesiology; *BMI* Body mass index

### Weight and BMI comparison of obese patients with ventral hernia

Significant differences were found in initial weight and BMI between the groups (*P* < 0.001). Bariatric surgery patients had the highest initial weight (116.9 kg) and BMI (42.8 kg/m^2^), followed by the pharmacotherapy group (99.1 kg, 37.9 kg/m^2^) and the dietary counseling group (95.0 kg, 35.5 kg/m^2^).

Bariatric surgery resulted in the greatest weight reduction, with a mean loss of 20.7% (SD: 15.5), compared with 4.6% (SD: 6.0) in the dietary counseling group and 4.4% (SD: 3.5) in the pharmacotherapy group (*P* < 0.001). BMI reduction followed a similar trend. Specifically, the BMI of the bariatric surgery group decreased from 42.8 kg/m^2^ to 33.9 kg/m^2^ (21.7% reduction). The BMI of the dietary counseling group decreased from 35.5 kg/m^2^ to 33.9 kg/m^2^ (5% reduction), and that of the pharmacotherapy group decreased from 37.9 kg/m^2^ to 36.9 kg/m^2^ (5.1% reduction) (Table 1).

### Surgical eligibility and adherence to the weight loss program in obese patients with ventral hernia

The proportion of patients proceeding to elective hernia repair was significantly higher in the bariatric surgery group (83%) than in the dietary counseling (44%) and pharmacotherapy groups (13%) (*P* = 0.001). The dietary counseling group had the highest dropout rate (21%), followed by the pharmacotherapy group (13%), while no patients dropped out in the bariatric surgery group (*P* = 0.173). During the preoperative period, seven adverse events (4%) were recorded, all within the dietary counseling group. These included five emergency surgeries due to acute hernia incarceration and two cases of skin complications requiring specific treatment and follow-up (Table 1).

### Surgical outcomes in patients with ventral hernia repair

A total of 165 patients underwent ventral hernia repair, including 92 nonobese patients, 52 with preoperative weight loss, and 21 without weight loss. There were no significant differences in age (mean 61.7 ± 13.5 years, *P* = 0.566) or sex distribution across the groups. However, BMI remained significantly higher in the no-weight-loss group (34.5 ± 5.5 kg/m^2^) compared with the weight-loss group (29.8 ± 2.6 kg/m^2^) and nonobese patients (26.4 ± 2.4 kg/m^2^) (*P* < 0.001) (Table 2).

**Table 2:** Characteristics of patients undergoing ventral hernia surgery

Variable	Total (*n* = 165)	Non-obese patients (*n* = 92)	Elective hernia repair with previous weight loss (*n* = 52)	Elective hernia repair without previous weight loss (*n* = 21)	P
Age (yrs) [mean (SD)]	61.7 (13.5)	62.5 (13.6)	60.1 (14.2)	62.3 (10.8)	0.566
Gender, *n* (%)					0.884
Male	94 (57)	52 (57)	29 (56)	13 (62)	
Female	71 (43)	40 (43)	23 (44)	8 (38)	
BMI (kg/m^2^) [mean, (SD)]	28.5 (4.1)	26.4 (2.4)	29.8 (2.6)	34.5 (5.5)	< 0.001
ASA score					0.643
I/II [*n*, (%)]	129 (78)	74 (80)	40 (77)	15 (71)	
III [*n*, (%)]	36 (22)	18 (20)	12 (23)	6 (29)	
Previous abdominal surgery, *n* (%)	128 (78)	70 (76)	44 (85)	14 (67)	0.219
Comorbidity, *n* (%)	141 (86)	81 (88)	42 (81)	18 (86)	0.493
Cardiovascular disease, *n* (%)	98 (59)	53 (58)	31 (59)	14 (67)	0.747
Chronic obstructive pulmonary disease, *n* (%)	11 (7)	7 (8)	4 (8)	0 (0)	0.423
Chronic nephropathy, n (%)	12 (7)	6 (7)	6 (12)	0 (0)	0.209
Liver cirrhosis, *n* (%)	5 (3)	2 (2)	2 (4)	1 (5)	0.755
Anticoagulant treatment, *n* (%)	9 (6)	6 (7)	2 (4)	1 (5)	0.785
Diabetes, *n* (%)	35 (21)	20 (22)	12 (23)	2 (10)	0.398
Active smoking, *n* (%)	29 (18)	19 (21)	7 (14)	3 (14)	0.505
Comorbidity more than one, *n* (%)	62 (38)	35 (38)	22 (42)	5 (24)	0.333
Recurrent hernia, *n* (%)	24 (15)	10 (11)	12 (23)	2 (10)	0.107
Defect width (cm) [mean (SD)]	5.4 (3.8)	5.4 (3.9)	6.1 (4.1)	3.9 (2.2)	0.086
Complex hernia, *n* (%)	89 (54)	45 (49)	31 (60)	13 (62)	0.342
Type of hernia [*n*, (%)]					0.510
Umbilical	43 (26)	25 (27)	10 (20)	8 (38)	
Midline	33 (20)	18 (20)	12 (22)	3 (14)	
Trocar	44 (27)	21 (23)	19 (36)	4 (19)	
Parastomal	3 (2)	1 (1)	1 (2)	1 (5)	
Combined	11 (7)	8 (9)	1 (2)	2 (10)	
Lateral	29 (17)	17 (18)	9 (18)	3 (14)	
Suprapubic	2 (1)	2 (2)	0 (0)	0 (0)	
Operative time (min) [mean, (SD)]	175.9 (96.8)	172.5 (103)	187.4 (91.3)	163.8 (81)	0.573
Surgical approach [*n*, (%)]					< 0.001
Open	128 (78)	68 (74)	47 (90)	13 (62)	
Laparoscopy	5 (3)	1 (1)	0 (0)	4 (19)	
Robotic	32 (19)	23 (25)	5 (10)	4 (19)	
Component separation [n, (%)]	24 (15)	14 (15)	8 (15)	2 (10)	0.783
Mesh position [*n*, (%)]					0.005
Onlay	17 (9)	5 (5)	8 (15)	1 (5)	
Inlay	1 (1)	1 (1)	0 (0)	0 (0)	
Retromuscular	141 (84)	82 (88)	43 (83)	16 (76)	
Sublay	6 (4)	2 (2)	0 (0)	4 (19)	
No mesh	3 (2)	2 (2)	1 (2)	0 (0)	
Postoperative complications [*n* (%)]	41 (25)	15 (16)	19 (37)	7 (33)	0.016
Clavien Dindo classification of postoperative complications [*n* (%)]					0.036
None	122 (74)	76 (83)	32 (62)	14 (67)	
I/II	39 (24)	14 (15)	18 (35)	7 (33)	
III	4 (2)	2 (2)	2 (3)	0 (0)	
Hematoma [*n* (%)]	11 (7)	2 (2)	6 (12)	3 (14)	0.031
Seroma [*n* (%)]	21 (13)	7 (8)	9 (17)	5 (24)	0.065
Wound infection [*n* (%)]	4 (2)	0 (0)	4 (8)	0 (0)	0.012
SSO [*n* (%)]	33 (20)	9 (10)	17 (33)	7 (33)	0.001
Medical complications [*n* (%)]	9 (6)	6 (7)	3 (6)	0 (0)	0.490
Length of stay (days) [mean (SD)]	3.4 (3.4)	3.1 (3.4)	4 (3.6)	3 (2.1)	0.210
Recurrence [*n*, (%)]	9 (6)	4 (4)	2 (4)	3 (14)	0.161

*BMI* Body mass index; *ASA* American Society of Anesthesiology; *SSO* Surgical site occurrences

The weight-loss group had a higher prevalence of recurrent hernias (23%) and larger mean defect widths (6.1 ± 4.1 cm) compared with nonobese patients (11%, 5.4 ± 3.9 cm) and the no-weight-loss group (10%, 3.9 ± 2.2 cm). Although most patients had at least one comorbidity (86%), multiple comorbidities were slightly more common in the weight-loss group (42%) than in nonobese patients (38%) and those without weight loss (24%), though these differences were not statistically significant (Table 2).

Postoperative complications were significantly higher in the weight-loss (37%, IC 95%: 23.5%–49.6%) and no-weight-loss (33%, IC 95%: 13.2%–53.5%) groups compared to nonobese patients (16%, IC 95%: 8.8%–23.9%) (*P* = 0.016). The most common complication was SSO, affecting 33% of patients in both the weight-loss and no-weight-loss groups, compared with 10% in nonobese patients (*P* = 0.001). Hematomas were more frequent in the weight-loss (12%) and no-weight-loss (14%) groups (*P* = 0.031), while surgical wound infections occurred exclusively in the weight-loss group (8%) (*P* = 0.012) (Table 2).

Recurrence rates were highest in the no-weight-loss group (14%, IC 95%: −0.7%–29.3%), though this difference was not statistically significant (*P* = 0.161). The weight-loss group had a recurrence rate of 4% (IC 95%: −1.4%–9.1%), while nonobese patients had a recurrence rate of 11% (IC 95%: 4.5%–17.2%). The mean follow-up period was 27 months (SD: 15.8).

## Discussion

This study evaluated the impact of different preoperative weight loss strategies on ventral hernia repair outcomes as well as postoperative results in patients with and without preoperative weight loss. Here, bariatric surgery emerged as the most effective method for achieving significant weight and BMI reductions. Nonobese patients experienced the lowest postoperative morbidity, whereas patients who lost weight preoperatively and those who did not exhibited comparable complication rates. These findings underscore the complexity of this patient population and suggest that weight loss alone may not suffice to fully mitigate surgical risks in certain contexts. **However,** the notable imbalance between intervention groups (particularly the smaller size of the pharmacotherapy and bariatric cohorts) limits the strength of direct comparisons. These differences likely reflect underlying variations in baseline characteristics, intervention intensity, and patient selection, and should be considered when interpreting results.

Although current guidelines and clinical consensus emphasize the importance of preoperative weight loss in obese patients undergoing ventral or incisional hernia repair, the optimal strategy to achieve this goal remains undefined [12, 18]. Our study compared three distinct approaches—dietary counseling, pharmacotherapy, and bariatric surgery—and revealed that bariatric surgery resulted in the most significant weight reduction (mean weight loss of 20.7%), which was significantly greater than that of dietary counseling (4.6%) and pharmacotherapy (4.4%). Although no prior studies have directly compared these three strategies in the context of ventral hernia repair, existing guidelines and expert recommendations recognize bariatric surgery as an effective option for achieving substantial and sustained weight loss in obese patients [18, 25]. Additionally, a significantly greater proportion of patients in the bariatric surgery group underwent elective hernia repair (83%), compared with 44% in the dietary counseling group and 13% in the pharmacotherapy group. This finding underscores the advantage of bariatric surgery in facilitating weight loss sufficient to meet surgical eligibility criteria. Moreover, the bariatric surgery group had no dropouts, highlighting the value of structured, multidisciplinary programs in supporting adherence [10]. However, bariatric surgery is not suitable for all patients, as it requires careful selection on the basis of clinical factors, patient preferences, and the availability of resources [18]. These findings suggest that although bariatric surgery may offer significant benefits for selected patients [25], other strategies remain important for individuals who are not candidates for surgical weight loss interventions.

Dietary and behavioral counseling, although less invasive, presents notable challenges. Although some studies have reported successful weight loss with this approach [26], it often delays hernia repair and depends heavily on patient motivation [27, 28]. Moreover, it had the highest dropout rate in our study (21%), likely due to low adherence, which may be influenced by factors such as difficulty maintaining lifestyle changes, lack of immediate results, and limited access to structured support programs. Additionally, this approach resulted in the lowest proportion of patients proceeding to elective hernia repair after weight loss (44%), highlighting its limited effectiveness in achieving surgical eligibility. Nonetheless, this strategy remains viable for patients with advanced age, significant comorbidities, or minimally symptomatic hernias, as well as those preferring a conservative approach. Multidisciplinary support, including regular follow-up with dietitians, psychologists, and primary care providers, has been shown to enhance adherence and improve long-term weight loss success [10, 29]. Pharmacotherapy for preoperative weight loss remains a relatively unexplored strategy in the context of ventral or incisional hernia repair. In our study, this approach resulted in modest weight reduction (4.4%) and the lowest rate of hernia repair after weight loss (13%), highlighting its limitations, which are likely influenced by adherence challenges and access barriers [13]. Common barriers to adherence in pharmacotherapy include cost, side effects, and lack of long-term insurance coverage, which may lead to discontinuation of treatment before achieving meaningful weight loss [14]. In our cohort, pharmacotherapy consisted exclusively of GLP-1 receptor agonists, a class of drugs that has shown efficacy in promoting weight loss and improving metabolic parameters [30]. Recent studies have evaluated the role of GLP-1 receptor agonists in preoperative weight loss for ventral hernia repair [14, 30]. Some have shown that while pharmacotherapy achieves weight loss comparable to dietary counseling, patients using GLP-1 receptor agonists reach surgical eligibility faster, allowing for earlier hernia repair [30]. Other studies comparing pharmacotherapy with bariatric surgery found that while bariatric surgery results in greater overall weight loss, GLP-1 receptor agonists enable patients to proceed to surgery more quickly, with similar perioperative outcomes [14]. These findings suggest that pharmacotherapy may accelerate preoperative optimization, potentially reducing delays in surgical intervention.

While our results align with these observations, our study provides a broader perspective by comparing three preoperative weight-loss strategies instead of two. Additionally, we found a lower surgical eligibility rate (13%) in the pharmacotherapy group, which contrasts with prior reports [14, 30]. This difference may be attributed to variations in patient selection, adherence challenges, or institutional protocols.

These findings reinforce that while GLP-1 receptor agonists may be a viable alternative for patients ineligible for bariatric surgery, their effectiveness depends on sustained adherence and structured multidisciplinary follow-up. Further prospective studies are needed to evaluate their long-term impact on surgical outcomes and recurrence rates. Although pharmacotherapy shows promise, its use should be individualized, with a focus on patients contraindicated for bariatric surgery or those preferring less invasive approaches, supported by multidisciplinary care to monitor adherence, adjust medications as needed, and address potential side effects.

Our findings are consistent with previous studies reporting that preoperative weight loss does not consistently reduce postoperative complication rates in ventral hernia repair [31]. Although our data suggest patterns consistent with prior research, these findings should be considered preliminary due to the small size of some subgroups and limited follow-up duration. Some authors attribute this to the rapid onset of weight loss, which can lead to nutritional deficiencies and impaired tissue quality, potentially increasing the risk of complications [28, 32]. In contrast, other studies suggest that preoperative weight loss may equalize outcomes between previously obese and nonobese patients [10]. In our study, patients who lost weight preoperatively had high complication rates (37%), similar to those who did not lose weight (33%) and significantly higher than those noted in nonobese patients (16%). Several factors may explain this finding. First, despite weight loss, these patients remained in the obese range (mean BMI of 30), suggesting that residual obesity continued to negatively impact outcomes. Second, the weight-loss group had a higher prevalence of recurrent hernias and larger defect widths, increasing surgical complexity. Third, rapid weight loss, particularly through bariatric surgery or pharmacotherapy, may contribute to nutritional deficiencies, which can compromise wound healing and tissue integrity [32]. Given that weight loss alone may not be sufficient to optimize outcomes, a comprehensive prehabilitation approach may be necessary. This should include preoperative nutritional screening and supplementation, metabolic optimization, targeted abdominal wall strengthening exercises, and individualized weight-loss strategies to ensure that patients achieve not only weight reduction but also adequate tissue quality and functional improvement [9]. Minimally invasive techniques were more frequently employed in the non-weight loss group than in those who lost weight or were not obese. This finding is explained by the adherence to clinical guidelines recommending the use of these approaches in these patients whenever possible to reduce surgical wound-related complications [33].

Hernia recurrence was lower in the weight loss group (4%) compared to the no weight-loss group (14%), suggesting a possible benefit of preoperative weight loss in reducing recurrence risk. However, this difference did not reach statistical significance, which may be partially explained by the relatively small sample size of the no weight-loss group (*n* = 21), limiting the statistical power to detect significant differences. While this trend could indicate a clinically relevant effect, larger studies are needed to confirm whether preoperative weight loss indeed reduces recurrence rates. Taken together, our results and the literature suggest that prehabilitation—structured interventions aimed at optimizing patients before surgery—should extend beyond weight reduction to include nutritional optimization [12]. Nutritional optimization focuses on ensuring adequate protein intake, micronutrient supplementation, and metabolic control to enhance tissue quality and wound healing [8]. Integrating these strategies into perioperative care may improve outcomes in this high-risk population [26].

This study has several limitations. First, its retrospective and single-center design inherently limits the ability to establish causality and generalize findings to broader populations. Institutional protocols, patient selection criteria, and surgeon preferences may have influenced the results, reducing their external validity. Future multicenter studies could strengthen these conclusions by incorporating standardized protocols across different settings and increasing sample size to improve statistical power. Second, the sample sizes within subgroups, particularly the pharmacotherapy and bariatric surgery groups, were relatively small and imbalanced compared to the dietary counseling group. This limits the strength of between-group comparisons and increases the risk of confounding due to differences in baseline characteristics or intervention intensity. Third, the lack of long-term follow-up prevents us from assessing the durability of the observed outcomes, such as recurrence rates and the impact of weight loss strategies on long-term surgical success. Fourth, patients in the weight-loss group retained a mean BMI of 30, reflecting residual obesity that may have influenced outcomes independently of preoperative optimization. Additionally, although weight-loss strategies were assigned based on clinical guidelines, patient preference influenced the final decision, potentially introducing selection bias. Factors such as motivation, access to care, and willingness to undergo intensive interventions may have affected outcomes. Finally, although the study focused on weight loss strategies, it did not comprehensively assess other components of prehabilitation, such as nutritional status or physical conditioning, which could further impact surgical outcomes. Future studies should incorporate standardized prehabilitation protocols, including objective nutritional assessments, evaluation of metabolic and inflammatory markers, and structured abdominal wall training programs. Additionally, long-term follow-up should evaluate not only recurrence and complication rates but also functional recovery and quality of life to provide a more comprehensive assessment of preoperative weight loss interventions. These limitations underscore the need for prospective, multicenter studies with larger cohorts and comprehensive evaluations to validate these findings and provide more robust evidence.

In conclusion, this study highlights the real-practice implementation of a structured prehabilitation pathway for obese patients undergoing ventral hernia repair. While bariatric surgery yielded the most substantial weight loss and highest surgical eligibility, all strategies presented unique challenges and limitations. Importantly, postoperative outcomes appeared influenced by residual obesity, hernia complexity, and preoperative risk profiles, suggesting that weight loss alone may be insufficient. These findings support the feasibility of individualized prehabilitation approaches and emphasize the need for prospective, multicenter studies to validate outcomes and optimize care for this high-risk population.
